# Editorial: The Therapeutic Potential of Transcranial Magnetic Stimulation in Addiction

**DOI:** 10.3389/fnins.2020.614642

**Published:** 2020-11-23

**Authors:** Marco Diana, Liana Fattore

**Affiliations:** ^1^“G. Minardi” Laboratory of Cognitive Neuroscience, Department Chemistry and Pharmacy, University of Sassari, Sassari, Italy; ^2^CNR Institute of Neuroscience-Cagliari, National Research Council, Rome, Italy

**Keywords:** drug addiction, gambling, neuromodulation, food addiction, TMS (repetitive transcranial magnetic stimulation), virtual reality

This Research Topic aims to draw the attention of researchers and medical staff to the promising evidence supporting the use of Transcranial Magnetic Stimulation (TMS) in addiction (Diana et al., 2017). A second aim is to promote a change in the way we consider addiction and, as a consequence, in the therapeutic approach of addicts (Fattore and Diana, 2016). Harm reduction has dominated the field in the last 40 years (Ritter and Cameron, 2006), but it would seem overwhelmed by current data indicating (1) an increment in the diffusion, marketing, and abuse of psychostimulants like cocaine and amphetamines (World Drug Report, 2020), a sector plagued by a lack of specific therapeutic tools, (2) appearance on the drug market of new psychoactive substances (NPS) like phenethylamines, synthetic cannabinoids and synthetic cathinones (Weinstein et al., 2017), and (3) the dramatic resumption of opioid abuse, as revealed by the overdose epidemic recorded in the US in the recent past (Lyden and Binswanger, 2019). All cues that call for a change and the need to involve the patient in the therapeutic path that (s)he him/herself has requested in order to obtain “the drug free” status (too often, in the past, portrtayed as an unattainable goal) (Peele, 2016).

We felt that the time has come to deepen and expand our understanding of the potential therapeutic effects of TMS in the treatment of addicts. We have invited leading groups of scientists working in the field to “make the point” on the effectiveness of TMS in addiction. As a result, the present Research Topic brings together 12 papers, namely one perspective study, two commentaries, two original articles, two clinical trials, two opinion articles, and three reviews of high quality and broad impact, which encompass commonly abused drugs, i.e., cocaine, amphetamine, methamphetamine, and alcohol, also including food addiction and gambling.

In their perspective study, Stramba-Badiale et al. propose and discuss the integration of TMS over the dorsolateral prefrontal cortex (dlPFC) with virtual reality (VR) food exposure as therapeutic interventions for food addiction. Indeed, increasing cortical activity through high-frequency rTMS over the left dlPFC and simultaneously improving the management of the emotional and behavioral component of craving in fully immersive VR environments is expected to reduce craving in patients with food addiction and consequent eating disorders. In the two commentaries to the paper by Quoilin et al. (2018) entitled “*Deficient Inhibition in Alcohol-Dependence: Let's Consider the Role of the Motor System!*”, Zhou et al. and Nardone et al. discuss the potential of motor cortex stimulation, which plays a pivotal role in the deficient inhibitory control, as a target site for intervention. In particular, Zhou et al. suggest that, since improved inhibitory control plays a significant role in preventing relapse in alcoholics, using TMS over the related motor cortex to modify inhibitory processes may be a prospective treatment for patients with addiction. Then, Nardone et al. note that altered motor cortical excitability may be caused not only by a dysfunction in the neural inhibitory (mainly GABA) circuits, but also by an impairment of the intracortical excitatory circuits and, therefore, also the excitatory (mainly glutamatergic) neurotransmission should be considered in alcohol-dependence.

The two original articles are both focused on the use of Intermittent Theta-Burst Stimulation (iTBS), a more tolerable protocol administered at lower intensities and shorter intervals than conventional repeated TMS (rTMS) protocols, as a treatment for cocaine use disorder (CUD). The paper by Sanna et al. shows the efficacy of iTBS of the PFC in reducing cocaine intake and craving in treatment-seeking CUD patients, with iTBS protocol being as efficacious as high-frequency stimulation in reducing cocaine intake and with dropouts and adverse side effects also being similar in the two protocols. Efficacy of iTBS on cocaine intake is supported by Steele et al. that show that accelerated iTBS to the left dlPFC administered in active, chronic cocaine users is both feasible and tolerable in actively using cocaine participants. Two further clinical studies analyzed the effect of TMS on other drugs of abuse, namely alcohol and methamphetamine. Specifically, Schluter et al. investigated the effects of add-on rTMS treatment on impulsivity measures in abstinent individuals under treatment for alcohol use disorder (AUD). Yet, contrary to the authors' hypotheses of a rTMS-induced increase in impulse control abilities, results suggest no additional effect of rTMS on impulsivity measures, suggesting that some protocol modifications could be necessary to bypass procedural limitations. The clinical efficacy and tolerability of intermittent and continuous theta burst stimulation protocols targeting left or right dlPFC on craving and mood changes in abstinent methamphetamine-dependent subjects are elegantly demonstrated in the study by Zhao et al..

Studies investigating the effects of TMS on cocaine, amphetamine, and methamphetamine craving are systematically illustrated by Ma et al. who in their systematic review and meta-analysis provide persuasive evidence for the feasibility of using high-frequency repetitive TMS to alleviate craving induced by dopaminergic drugs in chronic users. The rationale and potential for rTMS to treat cocaine and methamphetamine dependence are explained by Moretti et al. who reviewed findings from studies performed in healthy humans and animal models to identify and understand the neurobiological mechanisms underlying rTMS effects, with a focus on the dopaminergic and glutamatergic systems. Less robust, although promising, are findings in support of the effectiveness of TMS in treating gambling disorders, as illustrated by the systematic review by Zucchella et al. who points out the clinical and methodological heterogeneity of the studies performed so far and the need of methodologically sound, robust, and well-powered studies to reach reliable conclusions. In their opinion article, Spagnolo et al. propose combining pharmacotherapies, psychotherapies, and cognitive interventions (e.g., cognitive behavioral therapy) with neuromodulation interventions, since the state of the brain during the application of the stimulation may critically modulate the effects of brain stimulation and ultimately change treatment outcomes (state-dependency). Finally, Steele concludes by highlighting the importance of further evaluating the use of rTMS to treat not only cocaine, amphetamine, methamphetamine, and alcohol but also eating and gambling disorders.

It is worth noting how important it is to change the approach, also in the experimental design, and to shift from a “trial and error” observation, too often (ab)used in the past, to a “hypothesis-driven” approach, based on previous observations already acquired and confirmed in the field.

In this context, non-invasive techniques such as TMS and VR will likely help advance knowledge on their possible application as therapeutic options for addiction.

## Author Contributions

Both authors listed have made a substantial, direct and intellectual contribution to the work, and approved it for publication.

## Conflict of Interest

The authors declare that the research was conducted in the absence of any commercial or financial relationships that could be construed as a potential conflict of interest.

## References

[B1] Diana M. Raij T. Melis M. Nummenmaa A. Leggio L. Bonci A. (2017). Rehabilitating the addicted brain with transcranial magnetic stimulation. *Nat. Rev*. Neurosci. 18, 685–693. 10.1038/nrn.2017.113 28951609

[B2] Fattore L. Diana M. (2016). Drug addiction: an affective-cognitive disorder in need of a cure. Neurosci. Biobehav. Rev. 65, 341–361. 10.1016/j.neubiorev.2016.04.006 27095547

[B3] Lyden J. Binswanger I. A. (2019). The United States opioid epidemic. Semin. Perinatol. 43, 123–131. 10.1053/j.semperi.2019.01.001 30711195 PMC6578581

[B4] Peele S. (2016). People Control Their Addictions: No matter how much the “chronic” brain disease model of addiction indicates otherwise, we know that people can quit addictions - with special reference to harm reduction and mindfulness. Addict. Behav. Rep. 4, 97–101. 10.1016/j.abrep.2016.05.003 29511729 PMC5836519

[B5] Quoilin C. Wilhelm E. Maurage P. de Timary P. Duque J. (2018). Deficient inhibition in alcohol-dependence: let's consider the role of the motor system! Neuropsychopharmacology 43, 1851-1858. 10.1038/s41386-018-0074-0 29728650 PMC6046042

[B6] Ritter A. Cameron J. (2006). A review of the efficacy and effectiveness of harm reduction strategies for alcohol, tobacco and illicit drugs. Drug Alcohol Rev. 25, 611–624. 10.1080/09595230600944529 17132577

[B7] Weinstein A. M. Rosca P. Fattore L. London E. D. (2017). Synthetic cathinone and cannabinoid designer drugs pose a major risk for public health. Front. Psychiatry 8:156. 10.3389/fpsyt.2017.00156 28878698 PMC5572353

[B8] World Drug Report (2020). United Nations publication, Sales No. E.20.XI.6.

